# Commissioning of the Controlled and Automatized Testing Facility for Human Behavior and Control (CASITA)

**DOI:** 10.3390/s18092829

**Published:** 2018-08-27

**Authors:** Ignacio Rodríguez-Rodríguez, Aurora González Vidal, Alfonso P. Ramallo González, Miguel Ángel Zamora

**Affiliations:** Departamento de Ingeniería de la Información y las Comunicaciones, Facultad de Informática, Universidad de Murcia, 30100 Murcia, Spain; aurora.gonzalez2@um.es (A.G.V.); alfonsop.ramallo@um.es (A.P.R.G.); mzamora@um.es (M.A.Z.)

**Keywords:** modelling, energy, buildings

## Abstract

Human behavior is one of the most challenging aspects in the understanding of building physics. The need to evaluate it requires controlled environments and facilities in which researchers can test their methods. In this paper, we present the commissioning of the Controlled and Automatized Testing Facility for Human Behavior (CASITA). This is a controlled space emulation of an office or flat, with more than 20 environmental sensors, 5 electrical meters, and 10 actuators. Our contribution shown in this paper is the development of an infrastructure-Artificial Intelligence (AI) model pair that is perfectly integrated for the study of a variety of human energy use aspects. This facility will help to perform studies about human behavior in a controlled space. To verify this, we have tested this emulation for 60 days, in which equipment was turned on and off, the settings of the conditioning system were modified remotely, and lighting operation was similar to that in real behaviors. This period of commissioning generated 74.4 GB of raw data including high-frequency measurements. This work has shown that CASITA performs beyond expectations and that sensors and actuators could enable research on a variety of disciplines related to building physics and human behavior. Also, we have tested the PROPHET software, which was previously used in other disciplines and found that it could be an excellent complement to CASITA for experiments that require the prediction of several pertinent variables in a given study. Our contribution has also been to proof that this package is an ideal “soft” addition to the infrastructure. A case study forecasting energy consumption has been performed, concluding that the facility and the software PROPHET have a great potential for research and an outstanding accuracy.

## 1. Introduction

Energy is one of the most important topics of study worldwide. Most governments have implemented initiatives that aim at more energy-efficient societies because of an urgent need to decelerate (1) energy resources exhaustion and (2) greenhouse gas emissions. Buildings are responsible for up to 40% of the carbon emissions in developed countries [1]; it has been seen that their energy use can be reduced substantially not only via renovation of their thermal envelope [2,3] but also via the modification of users’ behavior [4].

This opportunity to reduce energy use via changes in behavior has come at the same time as a technological revolution. There now exist sophisticated information management systems to control the different working points of building infrastructures. These systems have already been proved to be effective solutions to the problem of high energy consumption associated with comfort spaces in buildings, see for example [4,5,6].

Energy consumption is a complex phenomenon in which many aspects play a role; only a comprehensive way of studying it can fully cover its social, economic, and behavioral aspects. The effectiveness of technological solutions for modifying human behavior seems to vary depending on the study [7,8]. It is for this reason that more experimental research on human behavior is needed. This is a topic that was being studied as early as 1960 when Newton et al. [9] outlined the difficulties of understanding human behavior in buildings. Today, there have been advances in the understanding of human behavior, such as the work of [10,11]. However, more testing is needed to continue improving this field of research. A research facility that can serve as a testing arena for this kind of experimentation with the control of all aspects of functioning systems in a building is highly valuable in this field.

The analysis of energy efficiency in built environments has received growing attention in the last decade [12,13,14]. One possible method to lower energy use could be to generate a management system to tackle this challenge. A home automation system based on the internet of things (IoT) can monitor and control intelligently the different infrastructures involved in a building’s energy consumption, while being able to provide comfort, security and communication, energy efficiency, and promote water, electricity, and fuel conservation. Hence, the research community is not only interested in the understanding and modeling of human behavior, but also on the developing and testing of control strategies for building automation based on IoT.

With respect to the advances in sensing and control infrastructure, the growth on information and communication technologies (ICT) offer an even greater potential in the near future [15], and has opened a door for considering homes as environments with many more devices (such as sensors controllers or actuators). A facility that serves to understand the interactions between humans and buildings will need to have all those components to perform valid research.

The internet of things represents a radical evolution of the current internet to a network of interconnected devices that not only harvest information from the environment (sensing), but also allow interacting, managing, and storing easily any kind of data [16,17,18]. Following an IoT approach, new home automation systems could allow fulfilling the requirements posed by the social changes and new trends in our way of life, facilitating the design of more human, personal, multifunctional, and flexible homes. This change seems to be coming soon as the European Commission has established that 16 of the European Union (EU) member states will implement a large-scale smart-meter rollout by 2020 [19].

The efficiency and accuracy of any home automation system is possible as far as good predictions can be achieved by developing models about the building status. Ergo, different variables have to be taken into account regarding their impact on the energy consumption of buildings, while attempting to consider them in an integral vision [20]. Making a suitable selection and analysis of them is not obvious. Not only do environmental parameters such as humidity and temperature have to be studied, others like human behavior, weather forecast, insulating materials, or thermal inertia should be also considered in order to obtain patterns that will make it possible to anticipate changes in order to avoid declines in comfortable conditions and rises in energy consumption.

With this purpose, the available data about a building and its context have to be interpreted to obtain valuable knowledge. Statistical and novel methods of data analysis allow researchers to establish correlations between variables and to generate performance models of a building, which can be used to ensure efficient responses by the automation system. Thus, in the context of data science, many new and more powerful technologies are bringing alternatives, or even breakthroughs, in the prediction of building energy consumption associated with thermal comfort [21].

The facilities we present for the Controlled and Automatized Testing Facility for Human Behavior (CASITA) have an IoT-based home automation system installed and operational, where experiments can be done in order to test human behavior and IoT solutions. It is located at the Technology Transfer Center of the University of Murcia, Murcia, Spain. This test lab has numerous sensors, actuators, and controllers providing data, which are able to be used to generate accurate models in order to predict energy consumption and many other variables related to building physics. In addition to this, we have coupled the software PROPHET as the soft component of the functioning of the infrastructure for variable forecasting and completion. In this work, two models of energy consumption forecasting will be presented and discussed.

For the commissioning of this infrastructure, a model of the energy consumption based on the novel PROPHET package has been developed within mathematical software R. It measures several variables and evaluates variables that are beneficial for weather forecasting, thereby filling the future time series of outdoor conditions to validate the infrastructure.

All the steps proposed in this paper describe how preliminary testing on the research facility was performed, which can be used to design efficient management systems for saving energy that are fully scalable and that can be applied with the same goal in other buildings with similar sensing and actuation levels. With this paper we contribute to the development of a facility that is pioneer according to the knowledge of the authors, as it sums up the IoT and hardware infrastructure to a soft facet consistent on algorithms of prediction included on the PROPHET library.

This paper is structured as follows: Section 2 describes the infrastructure. Section 3 describes the commissioning and a pilot study to verify the validity of the data and the analysis methods available in CASITA. Section 4 shows conclusions and further work, followed by the references.

## 2. The Controlled and Automatized Testing Facility for Human Behaviour (CASITA)

Currently, a smart building can be equipped with information and communication technology (ICT) systems, as can be seen in [22], where a sensor network is deployed in a house. Another example is shown in [23].

**Figure 1:**
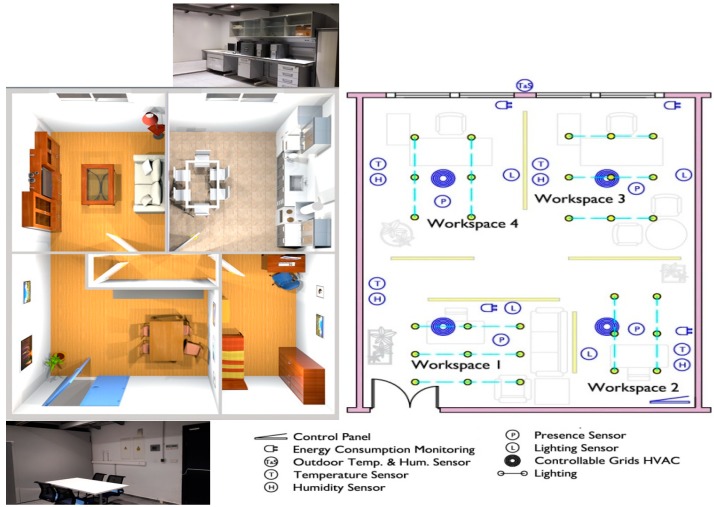
Infographic of a possible setup of the Controlled and Automatized Testing Facility for Human Behavior (CASITA) and distribution of all devices (sensors, actuators and controllers).

Although CASITA has been used before in other studies [6,24,25,26,27,28,29], the commissioning and description of the research facility had not been published yet. This paper aims to provide the necessary documentation to close this gap. CASITA (see Figure 1) is a case of a smart space with a wide deployment of sensors and devices integrated as if it was a home/office automation system.

In this highly sensed habitat, data referring to human behavior and to outdoor and indoor environmental parameters are collected.

### 2.1. Hardware

The home automation system installed in this reference scenario is composed by Programmable Logic Controllers (PLC), and a Supervisory Control and Data Acquisition (SCADA) system. This system has been given the name Domosec Platform [30]. All the sensors and actuators have been selected in accordance with the principles suggested on [31].

The PLC is able to monitor the sensor status and regulate the infrastructures connected to a platform, while the SCADA system collects data and intercommunicates with the PLC using the actuators. This platform has been designed and developed in-house and more information can be provided on request as it is open-source.

The indoor temperature, humidity, and luminosity are measured in several points of the space. This makes possible to have an idea of how homogeneous the conditions are across the monitored areas. Outdoor conditions are also registered by a weather station located on the top of the building.

Human behavior and presence sensors using passive infrared technology are present. The control access system is based on Radio Frequency Identification (RFID) technology (more details about the device deployment can be found in [6]. Systems and location are exposed in Figure 1.

Due to the importance of outside weather in the studies that are being carried out in CASITA (for example, to measure adaptive thermal comfort), its framework also counts on an ad hoc weather forecast algorithm based on Agencia Estatal de Meteorología (AEMET), the Spanish Meteorology Agency [32], but post-processed further to improve accuracy. This will be explained further in the following sections.

Regarding the actuators deployed in CASITA, there are two Heating, Ventilating and Air Conditioning (HVAC) systems installed in the ceiling that consist on an electric air-to-air heat pump (TOSHIBA RAV-SM803AT-E and 2xTOSHIBA SM806BT-E, Toshiba Carrier Corporation, Tokyo, Japan). Therefore, the indoor temperature and humidity can be modified in CASITA at the user’s will. The system has two levels of air velocity (fan power) and a thermostatic proportional control. The primary energy of the system is electricity.

**Figure 2:**
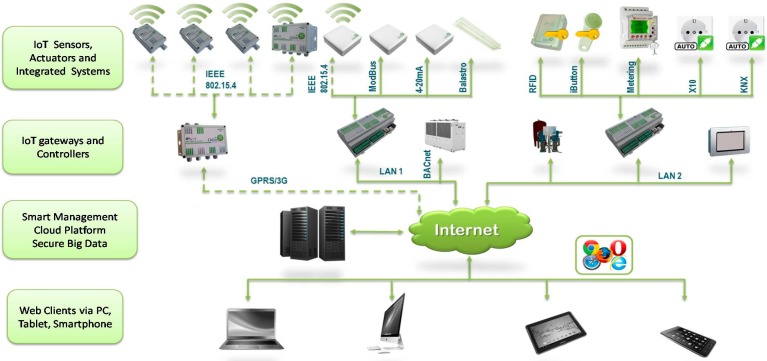
Hardware and communication architecture in CASITA.

**Figure 3:**
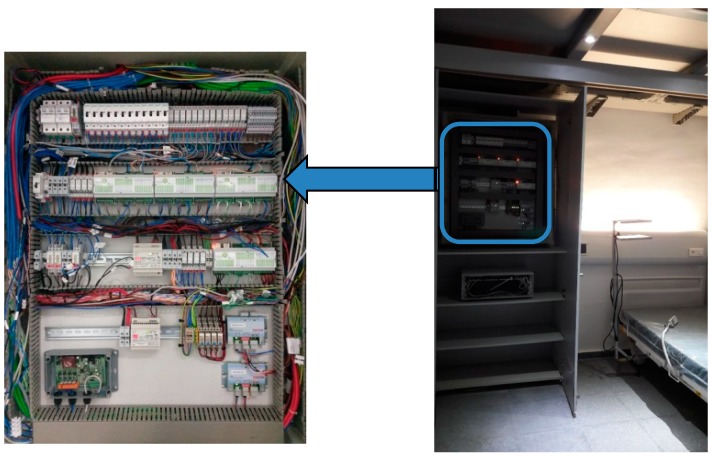
Wiring of data loggers, Supervisory Control and Data Acquisition (SCADA), and other devices in the main wiring cabinet of CASITA.

Lighting is provided via light-emitting diodes (LED) placed in the ceiling in accordance with current Spanish regulations. However, they are easy to move as the ceiling space is formed by removable panels. All lighting can also be controlled via the SCADA using the internet. A schema of the hardware and communication architecture in CASITA is shown in Figure 2, and the connection of all this equipment can be seen in Figure 3.

The electrical consumption of lights, HVAC units, and other electrical appliances are continuously being monitored and collected in the SCADA of the platform. Sensors can report at any given sampling period, which is at least 1 min long, but some of them are able to report at higher frequencies, e.g., high-frequency reporting of electrical grid to verify harmonics. Next table (Table 1) summarizes measured features and actuators that can be found in CASITA.

With this infrastructure, different choices can be combined in order to reach a goal of sufficient comfort, reduced energy consumption, combination thereof, or other objectives.

### 2.2. Software: The PROPHET Package

The PROPHET package is an utility to model time series and that serves as the perfect soft counterpart of the infrastructure shown in this paper. PROPHET is an R library that has been recently developed and seems to give promising results in other disciplines [34,35,36,37,38,39]. It is a modular regression model with interpretable parameters that can be intuitively adjusted with domain knowledge about the time series [40].

PROPHET conducts an automatic procedure for forecasting time-series data. The implemented algorithm uses Stan modelling language (allows to share the same core procedure between Python and R implementations) for optimization in order to fit a non-linear additive model and generate uncertainty intervals.

The additive regression model has four main components: a piecewise linear or logistic growth curve trend. Prophet detects changes in trends by selecting changepoints from the data, a yearly seasonal component modeled using Fourier series, a weekly seasonal component using dummy variables, and a user-provided list of important holidays. PROPHET is robust enough to address missing data, shifts in the trend, and typically handles outliers well.

It allows the prediction of a horizon of observations for a given time series that fulfills some characteristics that are common to the time series generated by human actions, where factors such as holidays could be known in advance.

In order to create the model, a decomposable time series model with three main model components will be used: trend, seasonality, and holidays. This is shown in Equation (1),

*y*(*t*) = *g*(*t*) + *s*(*t*) + *h*(*t*) + *ε*_*t*_, (1)

where:

- *y*(*t*): time series of interest.
- *g*(*t*): represents non-periodic components (using piecewise linear or logistic growth curve trend). PROPHET implements two trend models that cover many applications: a saturating growth model and a piecewise linear model with automatic change point selection.
- *s*(*t*): trend factor that represents periodic changes. Time series often have multi-period seasonality as a result of the human behaviors they represent. To fit and forecast these effects, we must specify seasonality models that are periodic functions of *t*. This part relies on Fourier series to provide a flexible model of periodic effects.
- *h*(*t*): effects of holidays (a list provided by the user). Holidays and events provide large, somewhat predictable shocks to many time series and often do not follow a periodic pattern, so their effects are not well modeled by a smooth cycle.
- *ε*_*t*_: error which will be assumed to follow a normal distribution.

This formulation is similar to a generalized additive model (GAM), a class of regression models with non-linear smoothers applied to the regressors. This approach has the advantage in that it decomposes easily and accommodates new components as necessary; for instance, when a new source of seasonality is identified. Thus, PROPHET frames the forecasting problem as a curve-fitting exercise which differs from the traditional models used for time series that account for the temporal dependence structure in the data: ARIMA This formulation provides several functional advantages with respect to ARIMA formulations: flexibility regarding seasonality with multiple periods, measurements do not need to be regularly spaced and missing values are handled, fitting is very fast, and the parameters of the forecasting model are easily interpretable [41].

## 3. Commissioning and Example of Data Analysis

For the commissioning of CASITA, we developed a test that involves the use of all of the main systems (sensors, meters, and actuators) that are found in CASITA. With this test, we verified the validity of the installations. We also made a valid test of a software package that has not been previously used for this purpose to forecast energy consumption. To do this, two models were built with the collected data. Their subsequent improvement became a topic of discussion as the inclusion of the weather forecast as a variable or not had to be determined.

The weather forecast was obtained from an official source (AEMET). The experiment consisted of generating simulated data of office use during 60 days from using the actuators, turning on and off equipment, and interfering with the conditioning system. This was done in an emulated manner to test the actuators and remote controllers of CASITA (all this was designed, run, and measured from an office 40 km away) and because it allowed us to access the ground truth. To ensure that other researchers interested in using CASITA would know appropriately what this facility has to offer, all of the data for this commissioning is available upon request.

**Figure 4:**
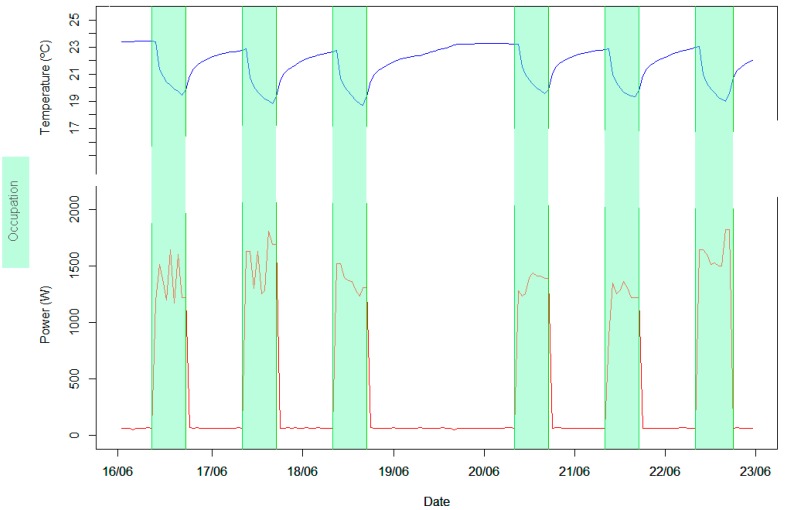
Representation of temperature and power for seven days of data in CASITA.

The experiment was conducted from 10 June to 14 August 2017. In this period, up to 4 workers were working in a normal schedule from 9:00 to 17:00. It is presumed that they developed their usual functions in an office environment, working at their desks, but also sometimes working in pairs or holding meetings all together. We do not consider metabolic activity of the workers or their humidity emission. Some workers had the possibility to work from home, so the number of people at the office fluctuated between 1 and 4 people; at other times, the place was empty (without air conditioning). The occupancy was registered from presence detectors and door-opening sensors, as well as energy consumption and distribution of the operating grilles and HVAC machines that were activated by employees on-demand. All data were collected hourly, even outside of the working schedule (24 h). The operating temperature of the HVAC machines was fixed to 20 °C. Representative variables of this experiment can be seen in Figure 4.

It is possible to appreciate that during the working time, energy consumption is triggered by the operation of office equipment air conditioning, which lowers the environmental temperature. In the graph, days are differentiated by higher or smaller occupancy and a local bank holiday, where no one was working at CASITA. Once the occupation is zero (in a working day at 17:00), it is easy to identify the fast rise of the indoor temperature due to the high temperatures outside. When night falls, the indoor temperature changes more slowly due to the lowering of the external temperature.

The aim of this verification is two-fold. First, we will evaluate the commissioning of CASITA, and second, as we have access to the ground truth of the test, we will verify the performance of PROPHET in the field of energy use prediction. We believe that if the results were positive, PROPHET could be used synergistically with CASITA for further research. We have aimed for a data-driven approach that does not take into account the physical properties of the building itself since it has been shown to be appropriate in similar scenarios [42]. Our models are used for predicting a horizon of energy consumption. This makes it different from other approaches [43], whose goal is the punctual prediction of a particular moment.

### 3.1. Verification of Accessible National Weather Forecasting in CASITA Using PROPHET

To make sure that CASITA offers well-tested weather data and weather forecasts, a stand-alone parallel study was performed.

For the start, it was necessary to study the relationship between the weather forecast obtained from the AEMET web page and the real outdoor conditions of our test lab. If the correlation between them was strong, it would be feasible to anticipate and predict the real outdoor conditions.

**Figure 5:**
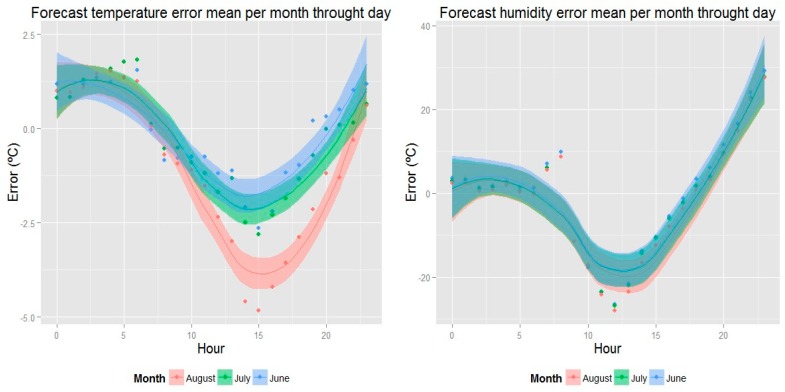
(**a**) Error mean between weather forecast (temperature) and outdoor temperature. (**b**) Error mean between weather forecast (humidity) and outdoor humidity.

In the case of an observed discrepancy between prediction and real data, steps were taken that allowed us to understand that error and to create a correction algorithm that reduces it substantially, adding value to the CASITA research facility. This is seen in Figure 5.

When one signal was subtracted from another, an error signal was obtained (see Figure 5), in which the mean square error per hour of this signal, organized by month, shows that the value of the discrepancy is predictable; this makes it possible to conclude that the weather forecast always has a similar lack of precision per hour, which can be modeled. We considered this an effect of the geographical surroundings of CASITA that are different to those of the location of the closest weather station of AEMET (Fuente Álamo).

**Figure 6:**
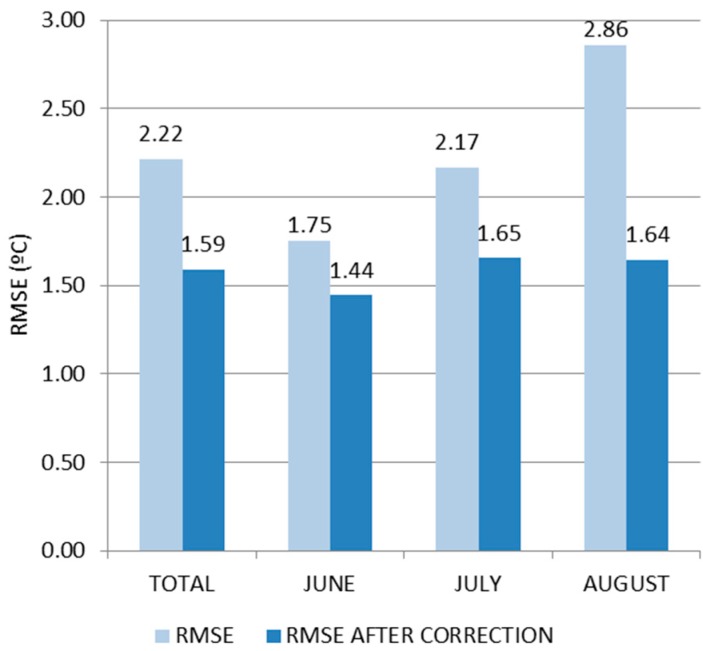
Root-mean-square error (RMSE) achieved after having into account the error mean evolution.

With this implemented, it is easy to introduce this correction into the weather forecast, and assess the achieved improvement that is related to the real outdoor conditions measured. As can be seen in Figure 6, the root-mean-square error (RMSE) has decreased substantially, (especially in August).

### 3.2. Validating Influence of the Variables Using PROPHET

**Figure 7:**
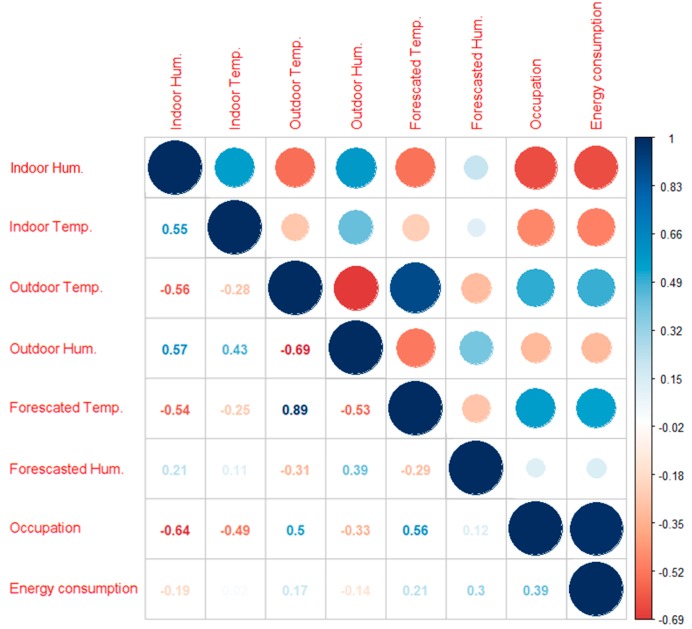
Cross correlation between influential variables in energy consumption. Done with a native routine in R: corrplot.

A preliminary study of the data was done to ensure that there were no missing values or misleading results that could crash a computer code for data analysis and to perform preliminary sanity checks. The corrplot routine was applied to relevant variables of energy use. And the results can be seen in the following (see Figure 7).

Once the calculation was done, it was learned that the correlation between conditions out of the building and inside are not very strong, which demonstrates that the conditioning systems work well, and the space is not sensitive to fluctuations on the weather outside. Indoor conditions are clearly affected by the HVAC operation, which is directly in relation to occupancy. In other words, the more people are working, the harder the air-conditioning devices are working; this is an expected result that demonstrates the validity of the data.

In one part of this preliminary study, we performed a validation exercise that uses the software package PROPHET. It is an open source code that runs in R and performs predictions of time series for many kinds of variables due to its large popularity in other disciplines; we thought it was interesting to test its performance in building physics.

Focusing on the variable that aimed to be forecasted, after studying the results it is possible to conclude that there is a correlation between energy consumption and indoor temperature and humidity. If one sees the results, outdoor temperature is an important variable, instead outdoor humidity is not that significant as one could expect. The same interpretation could be made regarding the weather forecast; temperature is relevant, and humidity is not. Occupation is revealed to be the more influential variable, as the presence of people is an essential requirement to have energy consumption.

In order to make a model of the energy consumption, there are some variables which show this to be influential:

- Occupation;
- Indoor conditions: temperature and humidity;
- Outdoor temperature;
- Forecasted temperature.

The energy consumption in buildings has several characteristics appropriate for the PROPHET algorithm and thus should perform well for energy prediction. These are:

- Strong multiple human-scale seasonality (such as day of the week and the time of year);
- Important holidays that occur at irregular intervals that are known in advance; and
- A certain random component.

Together with previous observations about energy consumption, in our problem the domain knowledge was defined by the inclusion of external regressors that were selected after observing the results:

Model 1: Forecasting energy consumption in a 24-h predictive horizon.

- Previous energy consumption.
- Previous occupation and future values of this variable with a known pattern and schedule.

Model 2: Forecasting energy consumption in a 24-h predictive horizon.

- Previous energy consumption.
- Previous occupation and future values of this variable with a known a pattern and schedule.
- Outdoor temperature values with temperature predictions filling the time series to be predicted.

**Figure 8:**
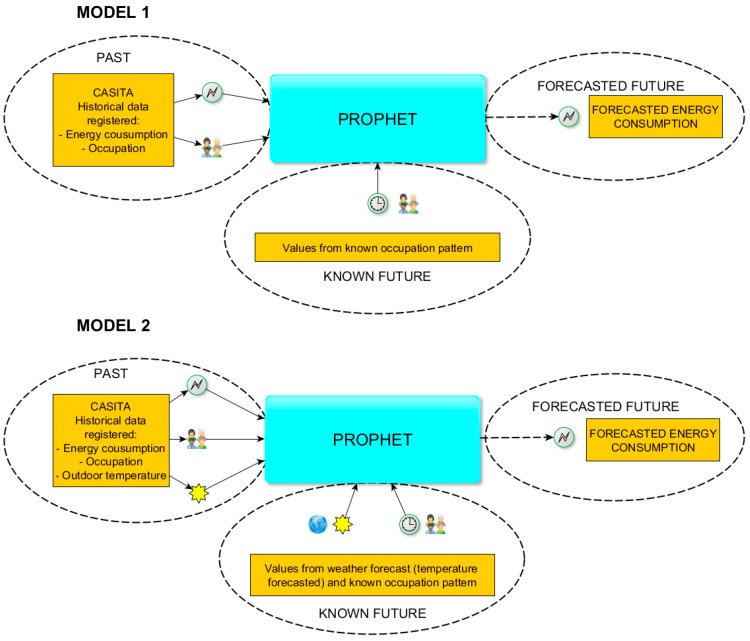
Schema of Model 1 and Model 2.

These models and their differences are explained in Figure 8. In Model 1 we forecast energy consumption only with previous values of this variable and previous occupation, completed with future occupation. Model 2 introduces, in addition, previous outdoor temperature measurements and the forecast are helped with future temperature values obtained from the national meteorological agency.

It was decided to perform the energy prediction using a sampling period of one hour, this was because that granularity captures most of the dynamics of the building without compromising the volume of data. An extra seasonality component was added that relates to the daily periodic of any energy-related variable linked to human behavior. The implementation was run on the R environment [41].

In order to make a first approximation, a prediction was performed for 12 August at 9:00, (start of working hours) with a predictive horizon of 24 h. In the following paragraphs, we studied two predictive models. These models are compared with the real measured energy consumption.

In the following points, two scenarios were tested, which contrasted with two different situations that provide the two different regressors previously mentioned.

**Figure 9:**
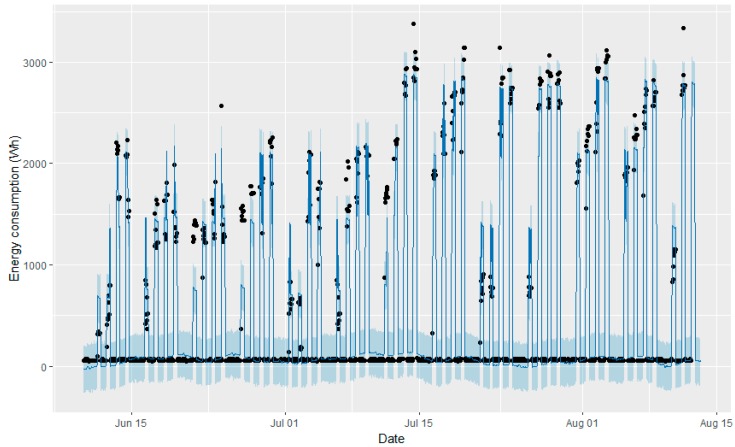
Twenty-four hour predictions performed with the fitter model (blue line) and the true values (black dots) with Model 1.

**Figure 10:**
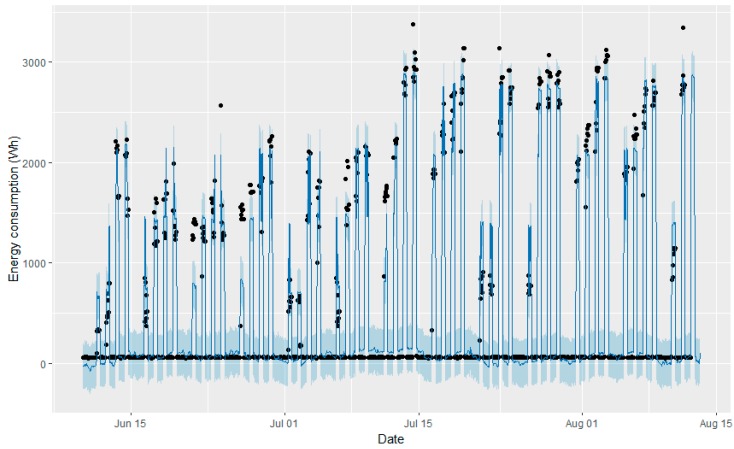
Twenty-four hour predictions performed with the fitter model (blue line) and the true values (black dots) with Model 2.

After running the models, it was possible to ask for the next 24-h prediction. Figure 9 and Figure 10 show the 24-h predictions performed with the fitter model (blue line) and the true values (black dots), while the last peak represents the forecasted energy consumption on 12 August, with a previously given occupation schedule.

**Figure 11:**
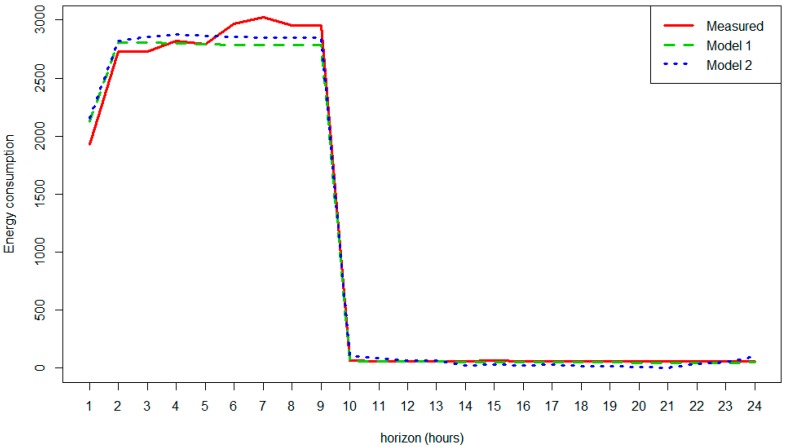
Energy consumption-real measures vs prediction (Model 1/Model 2).

Although both graphs seem to be very similar, the forecasted hours of the predictions slightly differ for the two models. These results serve as proof that both models have a good approximation to real measurements, but that there are some slight differences. It seems that Model 2 is closer to reality. A comparison between the real measures, and the forecasts with Model 1 and Model 2, is shown in Figure 11.

**Figure 12:**
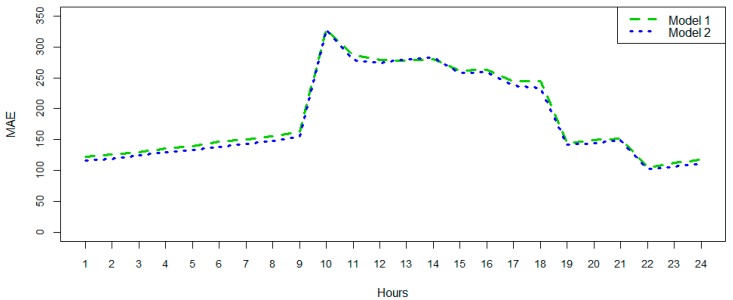
Mean absolute error (Model 1/Model 2).

After this example, and in order to evaluate this in a more comprehensive and general way, a cross validation was made to extract some conclusions of the different approaches. The cross validation assumes that these models could be generalized and that their accuracy of predictions estimated. In this iterative process, each hour is predicted using the rest of the available data, obtaining a measure of the error. This is done in Model 1 and Model 2 per hour several times. In addition, a visualisation of a given prediction is shown in Figure 12. It is then possible to estimate the mean absolute error (MAE) for each case under study by measuring the difference between the subsequent real measures and the predicted values. As we can see in Figure 12, combining the MAE per hour makes it possible to see that both have similar behavior. The MAE results in being higher in the working hours and are maximal at 9:00 when the HVAC starts; this is when the variability of the energy consumption is high and rapid. As is easily seen in Figure 12, Model 2 presents a better performance for nearly the entire day. Hence, the MAE is smaller and the accuracy of the model has been improved by adding outdoor temperature and temperature forecast in the prediction phase.

The improvement achieved can also be shown in terms of RMSE, offering an increase of accuracy. Global reduction of RMSE has been quantified in 4.54%, from the data exposed in Table 2.

## 4. Conclusions and Future Work

This work describes the commissioning of a new testing facility that has been given the name of Controlled and Automatized Testing Facility for Human Behavior (CASITA). The facility includes a large variety of sensors, meters, and actuators that allow the research to focus on fundamental aspects of the interactions of humans with built environments. The new contribution is that we have conceived this facility as a pair between the hardware and the software package PROPHET that provides the soft components (algorithms and analysis tools) to make the facility complete.

The first testing of this facility consisted of an occupation experiment that was performed to facilitate the posterior analysis of the software package PROPHET. The results of this software used in publications in other fields convinced us that it could be an excellent addition to CASITA for experiments that involved prediction (as there are many).

Building energy consumption models with new techniques are a of considerable interest to the scientific community. Our test experiment was to evaluate the functionalities of CASITA, and to ascertain the improvement of the PROPHET algorithms. Once the correlations were studied, two models were presented. After a brief explanation about a new tool for modeling and forecasting, the PROPHET package of the R software, some parameter settings and a comparison between the models became topics of discussion. The results indicate that introducing outdoor temperature into the model that uses the forecasted temperature provided by AEMET (an official source of weather forecast) improves the accuracy of its predictions.

The variables chosen in this work can be found in any residential or commercial building. As far as a sensor network that would be deployed, the same data can be collected and the models replicated. Therefore, the approach in this paper proposes an improved general model for forecasting energy consumption in buildings. A good approximation to this problem could enable one to plan for energy requirements, achieve energy and economic savings, and contribute to a more effective energy consumption policy.

In essence, the results show that CASITA is an excellent research facility that can be used for the testing of human modeling algorithms, IoT platforms, control strategies, and many more applications. With respect to the prediction algorithms tested here, both models are acceptable and achieve a good level of representativeness.

In addition, it is necessary to note that the algorithms tested have good accuracy but that they have not been compared with other methods, as the main aim of this work was commissioning CASITA and evaluating PROPHET as a side tool for it. We believe that the testing of its suitability was sufficient.

In future works, other scenarios will be tested. Weather forecasting will be added again along with other possible forecasted variables. We also plan to introduce human components into the equation, which would be interesting and will exploit the capabilities of CASITA well. In another vein, the PROPHET package became a tool whose benefits need to be studied further in this and other fields.

## Figures and Tables

**Table 1:** Description of the sensors and actuators available in CASITA.

Features	Sensor Deployments Allow Measurement of a Wide Set of Data
Weather data	Temperature and humidity.
Weather forecast	Up to 4 days.
Indoor conditions	In four different locations, temperature and humidity.
Occupancy and activity	A control access system in the test lab entrance and volumetric detectors in each room let predict in an accurate way the tracking of human presence.
Energy consumption:	For this purpose, and to monitor each component separately, non-intrusive load monitoring techniques have been considered [33]. We distinguish:
Electrical devices	Computers and other appliance are monitored.
Lighting	Differentiating each room.
Heating, Ventilation, and Air Conditioning (HVAC)	Each air-conditioned machine is quantified but is much bigger than the previous consumptions, which makes it energetically undesirable.
**Actuators**	**It is Possible to Modify the Test Lab Features, Comfort and Energy Consumption, Adapting the Next Actuators**
Access	Test lab can be completely locked, rendering it impossible to enter.
Control of the energy supplies	The plugs can be disabled completely.
Control of the HVAC machines	It is possible to force a shutdown or a start. The temperature set point and fan velocity mode can be chosen.
Ventilation grilles	Each air supply duct ends in a motorized ventilation grille (one per room), which can be opened or closed depending on the nature of its use in the area.

**Table 2:** Evolution of RMSE values over 24 h.

Hour	RMSE Model 1	RMSE Model 2	Improvement	Hour	RMSE Model 1	RMSE Model 2	Improvement
01	192.93	176.80	8.36%	13	378.35	384.33	−1.58%
02	200.24	182.96	8.63%	14	381.95	381.93	0.00%
03	210.39	191.86	8.81%	15	358.22	358.05	0.05%
04	222.05	202.28	8.90%	16	358.66	352.96	1.59%
05	231.73	212.67	8.23%	17	349.19	342.75	1.84%
06	243.96	222.99	8.59%	18	356.19	344.36	3.32%
07	251.60	230.77	8.28%	19	249.11	247.70	0.57%
08	262.76	239.10	9.00%	20	258.60	255.62	1.15%
09	275.33	250.72	8.94%	21	269.52	265.63	1.44%
10	427.56	432.00	−1.04%	22	160.57	149.72	6.75%
11	381.20	377.35	1.01%	23	172.48	159.43	7.57%
12	376.05	374.41	0.43%	24	181.99	167.22	8.12%
